# Estimating the subcritical transmissibility of the Zika outbreak in the State of Florida, USA, 2016

**DOI:** 10.1186/s12976-016-0046-1

**Published:** 2016-11-09

**Authors:** Linh Dinh, Gerardo Chowell, Kenji Mizumoto, Hiroshi Nishiura

**Affiliations:** 1 School of Public Health, Georgia State University, 1 Park Place, Atlanta, GA USA; 2 Division of International Epidemiology and Population Studies, Fogarty International Center, National Institutes of Health, Bethesda, MD USA; 3 Graduate School of Medicine, Hokkaido University, Kita 15 Jo Nishi 7 Chome, Kita-ku, Sapporo 060-8638 Japan; 4 CREST, Japan Science and Technology Agency, 4-1-8, Honcho, Kawaguchi-shi, Saitama 332-0012 Japan

**Keywords:** Prediction, Zika virus, Epidemic, Mathematical model, Basic reproduction number

## Abstract

**Background:**

Florida State has reported autochthonous transmission of Zika virus since late July 2016. Here we assessed the transmissibility associated with the outbreak and generated a short-term forecast.

**Methods:**

Time-dependent dynamics of imported cases reported in the state of Florida was approximated by a logistic growth equation. We estimated the reproduction number using the renewal equation in order to predict the incidence of local cases arising from both local and imported primary cases. Using a bootstrap method together with the logistic and renewal equations, a short-term forecast of local and imported cases was carried out.

**Results:**

The reproduction number was estimated at 0.16 (95 % Confidence Interval: 0.13, 0.19). Employing the logistic equation to capture a drastic decline in the number of imported cases expected through the course of 2016, together with the low estimate of the local reproduction number in Florida, the expected number of local reported cases was demonstrated to show an evident declining trend for the remainder of 2016.

**Conclusions:**

The risk of local transmission in the state of Florida is predicted to dramatically decline by the end of 2016.

## Background

The state of Florida has reported over 735 travel-related Zika cases since February 2016, becoming the first state in the continental USA to report multiple laboratory-confirmed autochthonous cases of Zika [1]. The state of Florida has not only experienced local transmission of Zika but also dengue and chikungunya viruses, which are transmitted via the common vector *Aedes* species.

To assess the risk of infection for local residents and travelers, it is critical to understand whether local transmission is sustained as well as forecast the duration and size of the outbreak. In this study, we carried out a risk assessment of Zika transmission in Florida aimed to estimate the extent of local transmission potential of Zika, e.g., are on-going local chains of transmission sustained in Florida? We also generated a short-term forecast of the expected burden of Zika for the remainder of 2016.

## Methods

### Patients’ data

In order to keep Florida residents and visitors abreast of the presence of Zika cases in the state, the Florida Department of Health has maintained up-to-date counts of the number of Zika cases diagnosed in this state [2] while strengthening a robust mosquito-borne illness surveillance system. Our study relies on confirmed cases of Zika virus infection. Before 29 June 2016, except for pregnant women, only those cases exhibiting at least two of the following symptoms: fever, rash, joint pain and red eyes and having an epidemiological link (travel history or sexual contact with travelers from Zika-affected areas or suspected contact with cases) underwent laboratory diagnosis by serology or rRT-PCR. From 29 June, all laboratory-confirmed asymptomatic cases were counted. Non-pregnant cases with recent travel history to an area with widespread Zika virus transmission were classified as either travel-related or non-travel related cases [3]. Hereafter, travel-related cases are referred to as imported cases, while non-travel related cases are referred to as local cases. We analysed the temporal evolution of confirmed Zika cases from 1 May to 23 September 2016. Since epidemiological and diagnostic procedures typically required 7 days [3], we analysed weekly case counts with week 0 starting on 1 May 2016.

### Modelling method

In order to quantify the extent of local transmission, we first set out to estimate the average and uncertainty of the reproduction number, *R*, associated with the Zika outbreak in the state of Florida. *R* is interpreted as the average number of secondary “local” transmission events caused by a single primary case. A primary case can be either a local or an imported case. Assuming that congenital or sexual transmission cases of Zika are rare in the state of Florida, our modelling exercise focused on mosquito-borne transmission alone. Let *c*_t_ and *i*_t_ be local and imported cases in week *t*, and let *w*_s_ represent the probability mass function of the serial interval of length *s* weeks, which was obtained by

1 $$ {w}_s=G(7s)-G\left(7\left(s-1\right)\right), $$

for *s* > 0 where *G*(.) represents the cumulative distribution function of the gamma distribution with mean of 14 days and standard deviation of 2 days [4]. We describe an expected value of local cases E(*c*_t_) as

2 $$ E\left({c}_t\right)={\displaystyle {\sum}_{s=1}^{\infty }R\left({c}_{t-s}+{i}_{t-s}\right){w}_s}, $$

as discussed elsewhere [5]. Assuming that variation in case counts in week *t* is sufficiently captured by the Poisson distribution, in agreement with the underlying mechanism of the infection process in deterministic models, the maximum likelihood estimate of *R* is obtained by minimizing the Poisson-distributed likelihood function that uses the right-hand side of (2) for the expected value. A constant *R* is supported by the negligible impact of herd immunity on the transmission dynamics due to the limited scope of the epidemic in this area thus far.

Subsequently, we assume that the dynamics of cumulative counts of imported cases, *I*(*τ*), at day *τ* is sufficiently captured by a logistic curve, i.e.,

3 $$ I\left(\tau \right)=\frac{K}{1+ \exp \left(-\gamma \left(\tau -{t}_0\right)\right)}, $$

where *K* is referred to as the carrying capacity (i.e., the expected total number of cases during the outbreak), *γ*, the steepness of the curve, and *t*_0_ the time of the sigmoid’s midpoint. In addition to the recent interest in using phenomenological models that generalize the logistic equation [6], the SIR (susceptible-infectious-recovered) model can be approximated by the logistic curve [7]. Hence, it may not be surprising that eq. (3) can be useful to capture single-epidemic transmission dynamics. Using (3), the expected weekly counts in week *t* was obtained by E(*i*_t_) = *I*(7 *t*)-*I*(7(*t* −1)) for *t* > 0. Assuming again that variation in imported case counts in week *t* is sufficiently captured by the Poisson distribution, maximum likelihood estimates of *K*, *γ* and *t*_0_ are obtained by minimizing the likelihood function that uses the right-hand side of (3) for the expected value. Profile likelihood was employed to compute the 95 % confidence intervals (CI).

Lastly, we employ a parametric bootstrap method to resample *R*, *K*, *γ* and *t*_0_ to generate short-term forecasts for case counts from week 21 (week starting with 25 September 2016) to 34 (week starting with 25 December 2016) [8]. The number of imported cases can be simulated using (3) and the number of local cases can be obtained by additionally using (2), accounting for the dependence among the estimated parameters. Posterior 95 % prediction intervals were derived by taking 2.5th and 97.5th percentile points from 1000 bootstrap simulations.

## Results

**Fig. 1:**
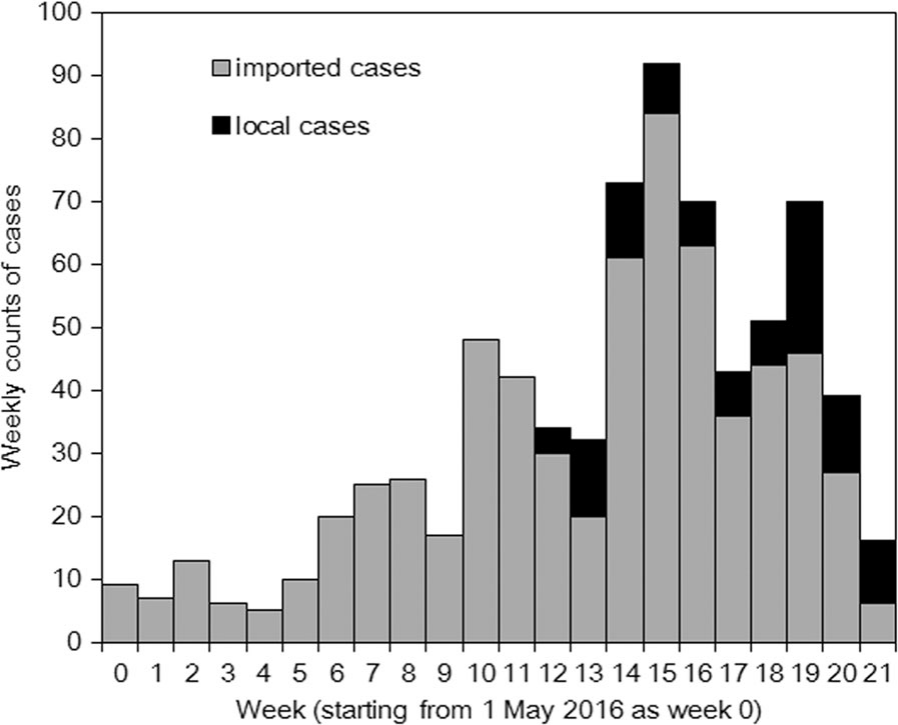
Observed weekly counts of confirmed cases of Zika virus infection, Florida, 2016. Imported cases have travel history to a country with widespread Zika virus transmission, while local cases were considered to arise from mosquito-borne transmission within Florida. Weekly counts start on 1 May 2016 and week 34 represents the last week of 2016

Figure 1 shows weekly case counts of reported Zika cases according to travel history in the state of Florida. A maximum count of 84 imported cases was observed in week 15 (week starting with 15 August), while the maximum count of 24 local cases was observed in week 19 (week starting with 11 September). The reproduction number *R* in Florida was estimated at 0.16 (95 % CI: 0.13, 0.19).

**Fig. 2:**
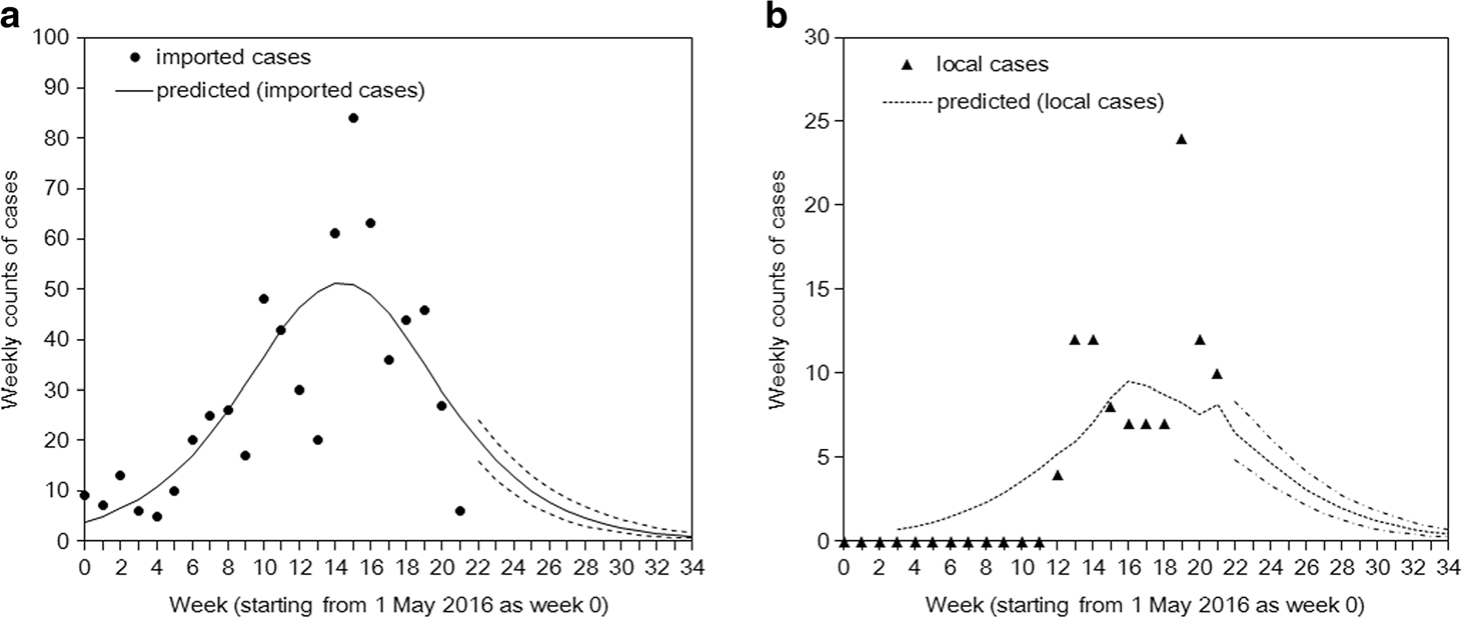
Observed and predicted time-dependent dynamics of Zika virus infection, Florida, 2016. **a**. Imported cases and **b**. local cases. Continuous line in **a** and dotted line in **b** represents the predicted results from maximum likelihood estimates of the reproduction number and parameters for logistic equation. Dashed lines in both panels from week 22 to 34 represent the prediction interval based on a Bootstrap method. Weekly counts start on 1 May 2016 and week 34 represents the last week of 2016

Figure 2a overlays the fit of our logistic model with imported cases. The cumulative number of imported cases was predicted to be 750 (95 % CI: 686, 823). As a function of time, the number of imported cases appears to be on a declining trend. From week 21 to 34 (i.e. remainder of 2016), we predict a total of 114 (95 % Prediction Interval (PrI): 85, 146) cases.

Figure 2b compares observed and predicted local cases of Zika virus infection in Florida. Due to the expected decline in the number of imported cases together with our low and subcritical estimate of the reproduction number that lies substantially below 1.0, the number of local cases are also expected to wane over time, with only a few additional cases expected towards the end of 2016. Our predicted number of additional imported cases from week 21 to 34 was estimated at 41 (95 % PrI: 29, 54) cases. It is worth noting that at the last time of writing, case counts for weeks 22–23, indicated an additional 36 imported and 33 local reported cases, which is in line with our forecast and the predicted decreasing trend in case counts.

## Discussion

In this study we analysed case series of reported cases of Zika virus infection in Florida State, May-September 2016, in order to estimate the local transmission potential and generate forecasts of the number of expected reported cases for the remainder of 2016. The reproduction number *R* of local Zika virus transmission in Florida was estimated to lie in the range of 0.13–0.19, likely reflecting primarily limited transmission potential and perhaps the effects of intensive vector control efforts including the strong advice of draining standing water, covering clothing and bare skin with repellent, and covering windows with screens. This finding indicates that local transmission chains in this area have not been sustained in the absence of continued importation of infected individuals. Moreover, the number of imported Zika cases has been declining since mid-August. If the declining trend continues for the remainder of 2016, our forecast based on the logistic growth model indicates a drastic decline in the number of imported cases expected by the end of 2016. This finding and our subcritical estimate of the local reproduction number in Florida point to a declining trend in the expected number of local reported cases for the remainder of 2016. Although the risk of local transmission within 2016 will not necessarily decline to zero, as transmission via the sexual route cannot be ruled out, the infection risk will be likely very small as the epidemic approaches the end of 2016. Although we employed a phenomenological model (e.g., did not explicitly account for seasonal mosquito population dynamics), our forecast may even be an overestimate as a significantly reduced risk of mosquito transmission can be expected during the winter season.

Our study is the first to have demonstrated that the effective transmission potential of Zika virus is substantially low in the state of Florida even in an area with a history of sustained transmission of dengue and chikungunya during the summer months. Our estimate of the local reproduction number at 0.16 is far below those estimates previously reported from areas with widespread Zika virus transmissions [9–12]. Reflecting the decline in the number of infections in Central America (e.g. Mexico) and South America (e.g. Colombia), the frequency of imported cases is also expected to decrease over time. At the same time, we have shown that local cases are also expected to decline [13]. With substantially decreased local transmission risk through the remainder of 2016, Florida could provide a basis for model-based transmission analyses and risk assessments of Zika across the world [14].

This study was conducted for real-time risk assessment of Zika virus infection, involving a number of important limitations. First, the dataset inherently involved reporting delays. It is likely that some symptomatic cases were retrospectively diagnosed by serology dating back to their date of illness onset. Rather than explicitly assessing the precise temporal transmission dynamics, we deemed sensible to generate an estimate of *R* based on a series of weekly case counts of limited precision. Second, asymptomatic transmission of Zika virus is known to be common [15], and the growing awareness of Zika virus among physicians is likely affecting differential diagnosis. Thus, observed confirmed cases must have experienced ascertainment bias. Third, the diagnostic criteria for reported cases confirmed with Zika was revised on 29 June 2016 to include asymptomatic individuals. This change was caused by the approval of a revised interim case definition in June 2016, which was originally issued in February 2016, along with the revision in their reporting system that partly relies on commercial laboratory testing. The ascertainment bias is likely to have been greatly reduced afterwards. Rather than attempting to provide a detailed analysis of the transmission dynamics of this outbreak using mechanistic models, we have intended to capture the overall temporal course of imported cases using a phenomenological approach. Fourth, our model did not explicitly account for the seasonality of local transmission. For this reason, our forecast for expected cases through the remainder of 2016 is likely an overestimate for a conservative assessment of the transmission risk associated with the outbreak.

While the revised version of this study was prepared in mid-October, a confirmed case was reported from Miami-Dade County. Despite possible transient increase in the number of cases, the expected decreasing trend of importation and limited transmission potential in Florida remains unchanged. Despite these limitations, we strongly believe that the present study sheds light on two critical issues, i.e. (i) transmission of Zika virus has not been locally sustained in Florida without continued external forcing and (ii) given a near-future reduction in imported cases, it is not farfetched to expect to dramatic decline in the number of local cases in Florida.

## Conclusions

A case series of reported cases of Zika virus infection in Florida State was analysed, to estimate the local transmission potential and generate forecasts of the number of expected reported cases for the remainder of 2016. The reproduction number *R* of local Zika virus transmission in Florida was estimated to lie in the range of 0.13-0.19, indicating that local transmission chains cannot be sustained in the absence of continued importation of infected individuals. Moreover, the number of imported Zika cases has been declining since mid-August. The expected number of local reported cases is very likely to show an evident declining trend for the remainder of 2016.

## Abbreviations

CI: Confidence interval
PrI: Prediction interval
rRT-PCR: Real-time reverse transcriptase polymerase chain reaction
